# Editorial: Reviews in veterinary neurology and neurosurgery

**DOI:** 10.3389/fvets.2025.1583995

**Published:** 2025-03-18

**Authors:** John H. Rossmeisl, Andrea Tipold

**Affiliations:** ^1^Department of Small Animal Clinical Sciences, Virginia Tech, Blacksburg, VA, United States; ^2^University of Veterinary Medicine Hannover, Hannover, Germany

**Keywords:** dyskinesia, epilepsy, hydrocephalus, meningoencephalitis, neuropathic pain

## Introduction

In recent years, the generation of knowledge in the field of veterinary and comparative neurology and neurosurgery has grown exponentially (1). The impact, diversity, and potential future applications of this knowledge are showcased in the 13 papers featured in this Research Topic. These papers address a variety of problems relevant to practitioners of veterinary neurology from common and long-recognized but persistently challenging conditions, such as chronic pain, cognitive dysfunction, epilepsy, and non-infectious meningoencephalitis, to more recently described diseases that include craniocervical junctional anomalies and paroxysmal dyskinesias, along with highly technically focused and specific reviews of the neurosurgical management of congenital malformations.

## Challenging the status quo: revising and refining diagnostic and management approaches for neuropathic pain, hemorrhagic CNS diseases and meningoencephalitis of unknown origin

Several articles in this Research Topic introduce new conceptual frameworks intended to facilitate the diagnosis, optimized management, or harmonized design of future studies of neurologic diseases. The contributions by Parker and Pedersen et al. address the complexities associated with the recognition and management of neuropathic pain in animals. These authors provide clinically relevant summaries of the form and function of nociceptive networks in health and disease and review advances in comparative pain research that collectively serve to improve the objective diagnosis of neuropathic pain, the rational selection of neuropathic pain modifying agents, and the development of new and effective analgesics. In their review of meningoencephalitis of unknown origin (MUO), Nessler and Tipold present a novel and holistic “polythetic” approach to the classification and treatment of MUO. Their proposal incorporates the heterogeneity and spectrum of clinical, neuroimaging, and immunopathologic manifestations of disease burden that may be present in each case in an effort to improve our understanding of the etiopathogenesis of MUO variants and to identify patient-specific therapeutic strategies. Santifort and Platt review the reported causes, treatments and outcomes associated with conditions presenting with intraparenchymal hemorrhage in the brain or spinal cord in humans, dogs, and cats, and subsequently propose a novel and succinct veterinary classification schema for hemorrhagic encephalopathies and myelopathies that synthesizes comparative, etiologic, and neuroanatomic localization data.

## Clinical applications of established and innovative technologies in veterinary neurology

The clinical utility of pervasive and emerging transformative technologies is illustrated in three papers included in this Research Topic. In their extensive review of paroxysmal dyskinesias (PD), Mandigers et al. highlight the value of phenotypic analyses of owner-acquired videos of paroxysmal events in conjunction with a focused historical probe and comprehensive clinical examination in the diagnosis and classification of canine PD. Robertson et al. provide an overview of the principles, promise, and limitations of a high throughput molecular analytical technique, Raman spectroscopy (RS), and demonstrate how RS-derived urine spectral fingerprinting can be used to identify and discriminate various etiologies of central nervous system disease in a rapid and economical manner. The article by Kim et al. describes the methodological development of a library of blood-based proteomic biomarkers of canine cognitive dysfunction, their validation against well-established mouse models of Alzheimer's and Parkinson's disease (2, 3), and the use of a machine learning analytical framework that revealed robust accuracies of the combination of retinol-binding protein 4 and NADPH oxidase 4 concentrations to clinically predict early neurobehavioral indicators of canine cognitive dysfunction.

## Epilepsy: lessons from the past, current concepts, and promises for the future

Three articles in this Research Topic focus on epilepsy, emphasizing both the progress that has been made in the diagnosis and treatment of this disease and the current challenges that veterinarians face when managing epileptic animals. Gouveia et al. provide a historical narrative and comprehensive multispecies review of the oldest known antiseizure drug, bromide, which is infused with numerous evidence-based and practical pearls of wisdom related to the indications, efficacy, toxicity, and nuances of therapeutic monitoring associated with bromide therapy. Articles by Foss and Billhymer and Kadler et al. provide state-of-the-art reviews on the established and evolving uses of qualitative and quantitative brain magnetic resonance imaging techniques in the context of epilepsy and highlight how these techniques have advanced the field of human epileptology, as well as the limitations that remain in incorporating these techniques into routine use in veterinary practice.

## Congenital malformations in companion animals

The final thematic area covered in this Research Topic relates to the diagnosis and treatment of congenital malformations affecting the brain, vertebral column, and spinal cord in dogs and cats. Schmidt and Farke provide a critical appraisal of the benefits, surgical techniques, and possible adverse events associated with ventriculoperitoneal shunting (VPS) for the treatment of internal hydrocephalus. This article includes excellent overviews of the diverse inventory of VPS instrumentation available for use, along with strategies for preventing and mitigating the range of complications that may be encountered in hydrocephalic patients treated with VPS. In their systematic review of the literature, Wess and Kneissl determine the prevalence of a spectrum of craniocervical junction abnormalities, such as occipital hypoplasia (OH), syringomyelia (SM) and atlanto-occipital overlap (AO) in dogs, and compare the frequency and potential clinical significance of these anomalies between dogs with and without a brachycephalic conformation. OH, SM, and AO were found to be significantly more likely to occur in small-breed, brachycephalic dogs, attributable clinical signs were infrequent in dogs with OH or AO that did not have contemporaneous SM, and only 1% of all dogs had concurrent imaging evidence of all three conditions. Roynard and Dewey summarize the currently available literature reporting on the neurosurgical management of lumbosacral meningomyelocele (MMC) in dogs, and contribute new clinical, diagnostic imaging, operative technical, and outcome data derived from the authors' experiences managing an additional nine dogs with MMC. Although the current body of literature reporting post-operative outcomes in dogs with MMC remains extremely limited, the work of these and other authors of recent studies suggests that early surgical intervention in dogs with MMC may result in improvement of pelvic limb neurologic function deficits and urinary and fecal incontinence in some dogs, and may prevent neurological deterioration associated with tethered cord syndrome (4).

The reviews included in this Research Topic provide a wealth of practical information on a wide range of subjects relevant to all levels of clinicians and researchers involved in the study or practice of veterinary neurology. To continue to generate rigorous evidence-based research and best practices for patient care, and to realize the potential of the tools and techniques covered in this Research Topic, it will be important for future studies to incorporate, critically evaluate, and subsequently refine the conceptual frameworks introduced here, and to apply and further validate these novel diagnostic technologies and therapeutic approaches in larger and more diverse animal populations.

## References

[1] Tipold A. Grand challenge veterinary neurology and neurosurgery: veterinary neurology and neurosurgery - research for animals and translational aspects. Front Vet Sci. (2015) 2:13. 10.3389/fvets.2015.00013 26664942 PMC4672178

[2] Boonpraman N Yoon S Kim CY Moon JS Yi SS. Nox4 as a critical effector mediating neuroinflammatory cytokines, myeloperoxidase and Osteopontin, specifically in astrocytes in the hippocampus in Parkinson's disease. Redox Biol. (2023) 62:102698. 10.1016/j.redox.2023.102698 37058998 PMC10123376

[3] Buxbaum J Roberts A Adame A Masliah E. Silencing of murine transthyretin and retinol binding protein genes has distinct and shared behavioral and neuropathologic effects. Neuroscience. (2014) 275:352–64. 10.1016/j.neuroscience.2014.06.019 24956283

[4] Martín Muñiz L Del Magno S Gandini G Pisoni L Menchetti M Foglia A. Surgical outcomes of six bulldogs with spinal lumbosacral meningomyelocele or meningocele. Vet Surg. (2020) 49:200–6. 10.1111/vsu.13342 31758707

